# Postacute Care Use and Outcomes Among Medicare Advantage vs Traditional Medicare Beneficiaries

**DOI:** 10.1001/jamanetworkopen.2025.40347

**Published:** 2025-10-29

**Authors:** Indrakshi Roy, Franya Hutchins, Liam Rose, Song Zhong, Syama R. Patel, Amit Kumar, Rachel M. Werner, Robert E. Burke

**Affiliations:** 1 Department of Epidemiology and Biostatistics, School of Public Health, Indiana University, Bloomington; 2 Regenstrief Institute, Indianapolis, Indiana; 3 Division of General Internal Medicine, University of Pennsylvania Perelman School of Medicine, Philadelphia; 4 Center for Health Equity Research and Promotion, Pittsburgh VA Medical Center, Pittsburgh, Pennsylvania; 5 Health Economics Resource Center, Palo Alto VA Medical Center, Palo Alto, California; 6 Stanford Surgery Policy Improvement Research and Education Center, Stanford University, Stanford, California; 7 Department of Physical Therapy, College of Health, University of Utah, Salt Lake City; 8 Center for Health Equity Research and Promotion, Corporal Crescenz VA Medical Center, Philadelphia, Pennsylvania; 9 Leonard Davis Institute of Health Economics, University of Pennsylvania, Philadelphia; 10 Division of Hospital Medicine, University of Pennsylvania Perelman School of Medicine; Philadelphia; 11 Associate Editor, *JAMA Network Open*

## Abstract

**Importance:**

Postacute care expenditures exceed $57.3 billion annually for traditional Medicare (TM) and drive regional spending variation. Medicare Advantage (MA) plans, with financial incentives to optimize postacute care, offer a compelling alternative. With more than half of Medicare beneficiaries now enrolled in MA, understanding postacute care use and outcomes across these groups is increasingly critical for policy and practice.

**Objective:**

To analyze the association of MA enrollment with postacute care use and patient outcomes compared with TM.

**Design, Setting, and Participants:**

This cross-temporal cohort study using a difference-in-differences approach matched 2021 MA beneficiaries to 2015 TM beneficiaries with a high propensity of enrolling in MA. The study included Medicare beneficiaries aged 66 years or older discharged alive from acute care in 2015 or 2021 and subsequently admitted to a skilled nursing facility (SNF) or to home health care. The data were analyzed between April 1, 2023, and August 28, 2025.

**Exposure:**

Enrollment in MA plans.

**Main Outcomes and Measures:**

The main outcomes were the proportion of beneficiaries discharged to an SNF or home health, length of stay in an SNF or home health, 100-day hospital readmission and mortality rates, total days in the community, and changes in functional status. Difference-in-differences analyses were conducted using linear probability models for binary outcomes, and linear regression models were used for continuous outcomes. Doubly robust models included the same covariates as the propensity score models to adjust for residual imbalances in the matching.

**Results:**

The study included 7 294 038 patients hospitalized in 2015 and 2021, with 2 687 009 (36.8%) enrolled in MA at some point. The final analytic sample included 1 081 103 MA beneficiaries enrolled in 2021 matched to 221 119 MA beneficiaries enrolled in 2015 (n = 1 302 222; mean [SD] age, 77.3 [7.9] years; 54.6% female) and 1 625 316 TM beneficiaries enrolled in 2021 matched to 534 607 TM beneficiaries enrolled in 2015 (n = 2 159 923; mean [SD] age, 78.4 [8.2] years; 53.9% female). The MA beneficiaries exhibited greater reductions in postacute care use compared with TM beneficiaries, including 6.3 fewer days in SNFs (95% CI, −6.8 to −5.8 days) and 3.6 fewer days in home health (95% CI, −4.3 to −2.9 days). Medicare Advantage enrollees also experienced a 1.5–percentage point lower probability of readmission (95% CI, −1.8 to −1.2 percentage points) and spent more time in the community in the first 100 days after hospital discharge (difference, 1.9 days; 95% CI, 1.7-2.2 days) than TM beneficiaries. Medicare Advantage beneficiaries also experienced a slightly lower mortality (difference, −0.3 percentage points; 95% CI, −0.6 to −0.1 percentage points) compared with TM beneficiaries, as well as modest functional gains (difference in 30-day activities of daily living improvement, 2.5 percentage points; 95% CI, 1.7-3.4 percentage points).

**Conclusions and Relevance:**

These findings suggest that reductions in postacute care in comparable MA and TM beneficiaries were not associated with worse outcomes.

## Key Points

**Question:**

Do postacute care use and outcomes differ between Medicare Advantage and traditional Medicare beneficiaries?

**Findings:**

This cohort study of 7 294 038 beneficiaries found that Medicare Advantage enrollment was not associated with postacute care setting decisions. However, Medicare Advantage enrollment was associated with fewer postacute care days, comparable mortality rates, and slightly greater functional improvements vs traditional Medicare.

**Meaning:**

These findings suggest that optimizing postacute care efficiency in traditional Medicare may reduce costs while maintaining beneficiary outcomes.

## Teaser

This cohort study examines the association of Medicare Advantage enrollment with postacute care use and patient outcomes compared with traditional Medicare.

## Introduction

More than 40% of traditional Medicare (TM) beneficiaries receive postacute care after hospitalization, primarily in skilled nursing facilities (SNFs) or through home health agencies.^1,2^ This care costs more than $57.3 billion annually.^3^ However, 25% of beneficiaries are readmitted to the hospital within 30 days, and nearly half of those in SNFs do not return to the community within 100 days.^4,5,6^ With Medicare Advantage (MA) enrollment surging from 19% in 2007 to 54% in 2024,^7^ a unique opportunity presents itself to explore alternative approaches to postacute care use and outcomes. Medicare Advantage plans operating under a capitated payment model emphasize cost efficiency through strategies such as prior authorization,^8^ restricted practitioner networks,^9^ and active care coordination to reduce lengths of stay.^10,11^ While these methods reduce costs and use, they raise concerns about potentially compromising long-term health outcomes for older Medicare beneficiaries. In particular, postacute care plays a critical role in the care trajectory of older adults following hospitalization, in which older adults may functionally recover and return to or remain home vs requiring more intensive long-term nursing home care.

Prior studies have suggested that MA and TM enrollees have similar outcomes despite decreased use of postacute care in MA.^12,13^ However, it has been difficult to address underlying differences in the MA and TM populations due to patient selection into their plan of choice. On the one hand, because MA plans receive capitated payments that are risk adjusted, MA plan insurers are incentivized to identify and code patient comorbidities,^14,15^ which may lead to MA beneficiaries appearing sicker in administrative data.^16,17^ On the other hand, MA beneficiaries often switch to TM after a health event, making the MA population appear healthier.^18,19,20,21^ Additionally, earlier studies often relied on limited data from a few selected MA insurers,^22^ studied a single state,^23^ used data predating 2015,^12^ or focused on specific clinical conditions.^13,24,25,26^ Thus, we sought to rigorously compare postacute care use in SNF and home health and outcomes (including hospital readmission, mortality, improvement in function, and time spent outside of institutional care settings) in the first 100 days following hospital discharge between similar MA and TM beneficiaries using a novel approach to help address potential selection.

## Methods

This cohort study was reviewed and considered exempt by the University of Pennsylvania Institutional Review Board as secondary use of identifiable private information per the revised Common Rule, exemption 4. As this study posed minimal risk, the requirement to obtain informed consent was waived as not practical to complete otherwise. The study followed the Strengthening the Reporting of Observational Studies in Epidemiology (STROBE) reporting guideline.^27^

### Data Sources

We used Medicare Provider Analysis and Review files, which contain 100% of Medicare beneficiaries’ inpatient stays, from 2015 and 2021 to identify short-term inpatient hospitalizations in acute care and critical access hospitals among Medicare beneficiaries. Postacute care stays were identified using the Minimum Data Set (MDS) 3.0 instrument for SNFs and the Outcome and Assessment Information Set (OASIS) instrument for home health to ensure inclusion of both MA and TM. Skilled nursing facility stays were defined as any admission date recorded in the MDS within 1 day of hospital discharge, as 97% of all SNF episodes in our sample occurred within this time frame.^28^ Home health episodes were identified using a 14-day window after hospital discharge.^29,30^ In cases of overlapping inpatient, SNF, or home health days, we applied a hierarchical rule prioritizing inpatient stay first, then SNF, then home health. If no discharge, hospital admission, or death date was recorded for an SNF or home health within 100 days, the beneficiary was assumed to have received that care for the full period.

### Study Population

The study population consisted of Medicare beneficiaries aged 66 years or older who had an inpatient admission in either 2015 or 2021. These beneficiaries were continuously enrolled in TM or MA for at least 1 year before hospitalization and for at least 100 days after discharge or until death.

We excluded hospitalizations of patients who died during their inpatient stay, remained hospitalized beyond 30 days, or were discharged against medical advice. Stays followed by inpatient rehabilitation or long-term acute care hospital admissions before SNF or home health were also excluded. Beneficiaries were categorized as MA or TM based on their Medicare Master Beneficiary Summary File coverage during the index hospitalization month. We excluded individuals who switched between TM and MA during the stay, were enrolled in the Program for Acute Care for the Elderly, or had private fee-for-service plans. Additionally, hospitalizations were excluded if county codes were missing or the county lacked MA plans at the time of admission. To ensure that postacute care choice was not influenced by prior postacute care or hospital use, we excluded hospitalizations preceded by another hospital stay, SNF stay, or home health episode within 100 days prior to index hospitalization. Full cohort creation details and exclusions and cohort descriptions are provided in eFigure 1 and eTable 1 in Supplement 1.

### Independent Variables

We described the MA and TM cohorts by demographics, including age, sex, and race and ethnicity (American Indian, Asian or Pacific Islander, Black, Hispanic, White, and other [missing or did not fall into the standard race categories reported in the Medicare Beneficiary Summary File] or unknown); dual Medicare-Medicaid eligibility status; and rural residence. Race and ethnicity were used as they have been shown to be associated with MA enrollment. Additional patient characteristics included a weighted Charlson Comorbidity Index (using diagnoses from the index hospitalization), a frailty index^31^ based on prior year data, hospitalizations in the past year, and the original reason for Medicare eligibility. At the county level, we included the number of Medicare beneficiaries, number of available MA plans, MA benchmark payment rates, MA plan quality (star rating), hospitals per 1000 beneficiaries, postacute care organization (SNF, home health care agency) per 1000 beneficiaries, and neighborhood disadvantage (assessed using the Area Deprivation Index).^32^

### Outcomes

We calculated the proportions of MA and TM beneficiaries hospitalized and receiving SNF or home health care in 2015 and 2021. Postacute use was measured by the proportion receiving care and total days used, summing all SNF or home health days from discharge to 100 days after discharge. Beneficiary outcomes over 100 days included hospital readmissions, mortality, and days spent in the community (ie, not in an SNF or inpatient care). The 100-day period was chosen because (1) most postacute episodes occur within 100 days; (2) Medicare’s SNF benefit is capped at 100 days per period, a benchmark for transition to long-term care; and (3) it balances very-short-term outcomes unlikely to be affected by postacute care with long-term outcomes for which the association between postacute care use and outcomes is less clear.

In addition to use outcomes, functional outcomes were measured as changes in activities of daily living (ADL) dependencies from admission to discharge during the first postacute care stay. This outcome period was chosen since most stays were 30 days or less. For residents not discharged within 30 days, the closest prior assessment was used as follow-up. Change in ADL was assessed using MDS or OASIS data, with bathing, dressing, toileting, transferring, and eating dichotomized by independence or need for assistance. Urinary and bowel incontinence were categorized as always continent (or with catheter or ostomy) vs not always continent. A summed score of these 7 items was calculated at entry and follow-up, with follow-up defined as the discharge assessment for residents discharged within 30 days from an SNF or home health. Residents with lower follow-up scores compared with the admission assessment (ie, fewer ADL dependencies) were considered to have improved.^33^ Functional status was evaluated and compared within each setting (ie, functional status change was measured separately from SNF admission to 30 days later for beneficiaries discharged to an SNF and from admission to 30 days later for beneficiaries discharged to home health).

### Conceptual Approach to Analysis

An estimate of the association of MA enrollment with SNF and home health use and patient outcomes is vulnerable to 2 major sources of bias: (1) differences in the underlying populations of beneficiaries who chose TM vs MA and (2) change over time in trends of SNF and home health use. Propensity matching is a common tool to address the first source of bias, and a difference-in-differences approach was used to address the second. However, neither alone address both. Instead, we took advantage of the substantial growth in MA enrollment (from 32% in 2015 to 46% in 2021^7^) and applied a novel cross-temporal matching design with difference-in-differences.^34,35^

Cross-temporal matching improves on standard propensity matching, which would otherwise pair 2021 MA enrollees with similar 2021 TM beneficiaries. Instead, cross-temporal matching identifies 2021 MA enrollees who resembled 2015 TM beneficiaries, generating a group that likely would have remained in TM if not for the rapid growth of MA enrollment nationally. This MA growth–matched group was then compared with matched TM beneficiaries in a difference-in-differences design. This approach improves the likelihood of identifying TM matches who more closely resemble the types of individuals who enrolled in MA by 2021, thereby reducing bias due to changing enrollee composition over time. Prior studies have used this approach to examine outcome changes associated with large secular trends over time.^34,35,36,37^ We discuss this method in more detail along with requisite assumptions in the eAppendix in Supplement 1.

### Propensity Matching

We used 2 logistic regression models to estimate MA enrollment in 2015 and 2021 (C statistic, 0.66 and 0.72, respectively). Matching was performed 1:1, with replacement using nearest-neighbor scores. This process created 3 groups. Group 1 included the always-MA beneficiaries (always-takers) who were enrolled in MA in both 2015 and 2021. Since group 1 was always exposed to MA, these beneficiaries were excluded from the main analyses. Group 2 was our exposed group (or compliers), which included beneficiaries who enrolled in MA in 2021 but TM in 2015 and were most likely to switch due to changes in availability of MA. Group 3 was our unexposed group (never-takers), which included beneficiaries who were in TM in both 2015 and 2021. All 3 groups were matched across years to capture secular trends (Figure). For ease of discussion, we refer to group 2 as MA beneficiaries and group 3 as TM beneficiaries. A flowchart describing the cross-temporal matching process is presented in eFigure 2 in Supplement 1, and the covariate balance is shown in eTables 2 and 3 and eFigure 3 in Supplement 1.

**Figure.**
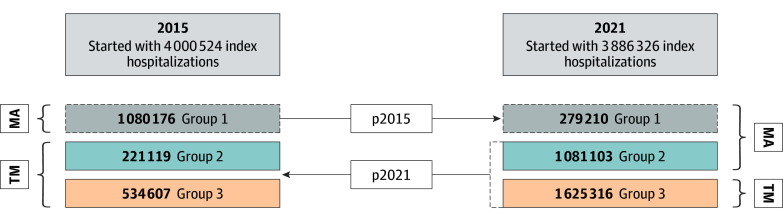
Cross-Temporal Matching Design For the cross-temporal matching process, in step 1, we estimated a propensity score for Medicare Advantage (MA) use with our 2015 baseline cohort (p2015). In step 2, to construct our treatment group 2, we estimated a propensity score with the 2021 postbaseline cohort (p2021). These steps are explained in detail in the eAppendix in Supplement 1. TM indicates traditional Medicare.

### Statistical Analysis

#### Main Analysis

The data analysis was performed between April 1, 2023, and August 28, 2025, using SAS, version 9.4 (SAS Institute Inc) and RStudio Pro, version 2023.12.1, build 402.pro1 (Posit Software, PBC). The threshold for significance was *P* < .05.

We assessed the balance in observed characteristics between the 2 groups by calculating standardized mean differences between unmatched and matched groups. Using the cohorts created by cross-temporal matching, we conducted a difference-in-differences analysis using linear probability models for binary outcomes, such as discharge to SNF, discharge to home health, readmission rate, and mortality rate. For continuous outcomes such as the number of days in an SNF, days in home health, and days in the community, we applied linear regression models. Doubly robust models included the same covariates as the propensity score models to adjust for residual imbalances in our matching. We estimated robust SEs to account for clustering of patients within counties.

#### Sensitivity Analyses

Since 2021 may reflect pandemic-related disruptions in health care delivery, we reestimated our models using 2015 to 2019 data (eTables 4 and 5 in Supplement 1). Additionally, to ensure that composition of hospitalizations did not change differentially for MA and TM groups between 2015 and 2021, we described the distribution of the top 10 diagnosis-related group codes across 2015 and 2021 to see whether we notice differential patterns of hospitalization in MA vs TM (eTables 6 and 7 in Supplement 1).

## Results

### Beneficiary Characteristics

Our cohort included 7 294 038 patients hospitalized in the preperiod (n = 3 704 118) or postperiod (n = 3 589 920), with 2 687 009 (36.8%) enrolled in MA at some point. Using our matching approach, we matched 1 080 176 MA beneficiaries enrolled in 2015 to 279 210 MA beneficiaries enrolled in 2021 and excluded this group (group 1) (Figure). The final analytic sample included 1 081 103 MA beneficiaries enrolled in 2021 matched to 221 119 MA beneficiaries enrolled in 2015 (MA group) (n = 1 302 222; mean [SD] age, 77.3 [7.9] years; 54.6% female and 45.4% male; 0.2% identifying as American Indian, 3.2% as Asian or Pacific Islander, 16.5% as Black, 12.0% as Hispanic, 66.4% as White, and 1.8% as other or unknown race and ethnicity) and 1 625 316 TM beneficiaries enrolled in 2021 matched to 534 607 TM beneficiaries enrolled in 2015 (TM group) (n = 2 159 923; mean [SD] age, 78.4 [8.2] years; 53.9% female and 46.1% male; 0.6% identifying as American Indian, 2.0% as Asian or Pacific Islander, 5.9% as Black, 3.0% as Hispanic, 86.4% as White, and 2.1% as other or unknown race and ethnicity). The largest residual differences after matching were primarily county-level characteristics and race and ethnicity (Table 1). As might be expected, MA beneficiaries had more locally available MA plans and higher quality plans than TM control beneficiaries. The MA group also had higher proportions of racial and ethnic minority beneficiaries and dual Medicare and Medicaid enrollees. Otherwise, the standardized mean differences within matched groups were less than 0.10 for frailty score, Charlson Comorbidity Index, and prior hospitalization (Table 1).^38,39^

**Table 1.** Propensity-Matched Sample for Difference-in-Differences Analysis

Covariate	MA (group 2) (n = 1 302 222), No. (%)^a^		Absolute SMD	TM (group 3) (n = 2 159 923), No. (%)^a^	
	2015 (n = 221 119)	2021 (n = 1 081 103)		2015 (n = 534 607)	2021 (n = 1 625 316)
Age, mean (SD), y	76.3 (7.9)	77.5 (7.8)	0.16	78.1 (8.2)	78.2 (8.2)
Sex					
Female	127 829 (57.8)	582 715 (53.9)	0.08	300 182 (56.2)	863 530 (53.1)
Male	93 290 (42.2)	498 388 (46.1)	0.08	234 425 (43.9)	761 786 (46.9)
Race and ethnicity					
American Indian	88 (<0.1)	1946 (0.2)	0.04	1978 (0.4)	10 564 (0.7)
Asian or Pacific Islander	5638 (2.6)	36 433 (3.4)	0.05	12 723 (2.4)	30 231 (1.9)
Black	75 313 (34.1)	139 030 (12.9)	0.52	53 621 (10.0)	73 952 (4.6)
Hispanic	55 657 (25.3)	100 326 (9.4)	0.43	27 800 (5.2)	36 245 (2.2)
White	83 428 (37.7)	781 421 (72.3)	0.74	432 604 (80.9)	1 434 504 (88.3)
Other or unknown^b^	995 (0.5)	21 947 (2.0)	0.14	5881 (1.1)	39 820 (2.5)
Dual Medicare-Medicaid enrollment					
Yes	96 496 (43.6)	226 167 (20.9)	0.50	105 959 (19.8)	195 526 (12.0)
No	124 623 (56.4)	854 936 (79.1)	0.50	428 648 (80.2)	1 429 790 (88.0)
Weighted Charlson Comorbidity Index, mean (SD)^c^	2.98 (2.53)	2.79 (2.48)	0.04	2.31 (2.25)	2.50 (2.38)
Frailty score, mean (SD)^d^	0.18 (0.04)	0.18 (0.04)	0.04	0.18 (0.04)	0.18 (0.04)
Area deprivation index, mean (SD)^e^	56.9 (18.7)	49.4 (20.7)	0.38	52.8 (20.5)	50.5 (21.9)
Medicare population in beneficiary county, mean (SD), No.	131 903.2 (138 008.5)	216 528.3 (299 592.2)	0.36	108 037.6 (136 729.2)	136 211.3 (226 653.1)
Availability of MA plans in beneficiary county, mean (SD), No.	21.2 (11.0)	77.6 (23.2)	3.12	17.0 (11.1)	57.1 (27.1)
Benchmark rate, mean (SD), $	764.0 (41.1)	979.4 (43.0)	5.12	770.6 (47.6)	987.1 (46.5)
MA plan rating in beneficiary county, mean (SD)^f^	3.80 (0.02)	3.91 (0.03)	4.77	3.80 (0.02)	3.91 (0.02)
Hospitals per 1000 beneficiaries in county, mean (SD), No.	0.19 (0.15)	0.16 (0.12)	0.18	0.24 (0.29)	0.20 (0.24)
SNFs or HHAs per 1000 beneficiaries in county, mean (SD), No.	1.04 (0.77)	0.84 (0.56)	0.30	1.29 (1.63)	0.87 (0.66)
Rural residence	6810 (3.1)	75 029 (6.9)	0.17	77 197 (14.4)	328 476 (20.2)
Original Medicare eligibility due to age	155 292 (70.2)	896 451 (82.9)	0.30	454 790 (85.1)	1 427 515 (87.8)
Prior hospitalizations in the year before index acute stay					
0	183 905 (83.2)	898 721 (83.1)	0.00	425 601 (79.6)	1 320 407 (81.2)
1	27 043 (12.2)	128 543 (11.9)	0.01	75 593 (14.1)	209 828 (12.9)
2	6987 (3.2)	35 136 (3.3)	0.01	21 598 (4.0)	60 462 (3.7)
≥3	3184 (1.4)	18 703 (1.7)	0.02	11 815 (2.2)	34 619 (2.1)

Abbreviations: HHA, home health agency; MA, Medicare Advantage; SMD, standardized mean difference; SNF, skilled nursing facility; TM, traditional Medicare.

^a^ Values reflect the composition of the matched analytic samples used to ensure comparability between MA and TM groups. They do not represent the overall demographic distribution of Medicare beneficiaries in 2015 or 2021.

^b^ Other race refers to beneficiaries whose race was either missing or did not fall into any of the standard race categories reported in the Medicare Beneficiary Summary File.

^c^ On a scale from 0 to 29, with higher values indicating greater morbidity.

^d^ On a scale from 0 to 1, with higher values indicating greater frailty.

^e^ On a scale from 1 to 100, with higher values representing greater levels of deprivation and reduced access to resources.

^f^ On a scale from 1 to 5, with higher values indicating better ratings.

### Postacute Care Use

Discharge to an SNF and home health declined in both the MA and TM groups between 2015 and 2021 (Table 2). Difference-in-differences estimates indicated no significant difference in probability of any postacute care use between MA and TM (difference, 0.6 percentage points; 95% CI, −0.1 to 1.2 percentage points). The MA group had a 0.8–percentage point higher probability of SNF use (95% CI, 0.4-1.2 percentage points) compared with TM, but home health use was not significantly different (difference, −0.3 percentage points; 95% CI, −0.8 to 0.3 percentage points). Mean length of stay in an SNF and home health decreased between 2015 and 2021, with a larger decrease among MA beneficiaries by 6.3 days (95% CI, −6.8 to −5.8 days) in an SNF on a basis of 35.5 days and 3.6 days (95% CI, −4.3 to −2.9 days) in home health on a basis of 33.6 days compared with TM beneficiaries.

**Table 2.** Observed Rates and Estimated Associations of MA With Postacute Care Use and Outcomes (n = 3 462 145 Hospitalizations)^a^

Outcome	Treatment group MA, No. (%)		Control group TM, No. (%)		Estimated association of MA, percentage points (95% CI)^b^	
	2015	2021	2015	2021	Unadjusted	Doubly robust adjusted
No. of beneficiaries	221 119	1 081 103	534 607	1 625 316	NA	NA
Any SNF	51 861 (23.5)	174 847 (16.2)	124 328 (23.3)	253 571 (15.6)	0.4 (−0.2 to 0.9)	0.8 (0.4 to 1.2)
Any HHA	34 166 (15.5)	137 721 (12.7)	81 845 (15.3)	217 351 (13.4)	−0.5 (−1.0 to 0.1)	−0.3 (−0.8 to 0.3)
Any postacute care	85 964 (38.9)	312 209 (28.9)	206 000 (38.5)	470 414 (28.9)	−0.1 (−0.8 to 0.6)	0.6 (−0.1 to 1.2)
Readmission	58 998 (26.7)	246 469 (22.8)	130 961 (24.5)	376 066 (23.1)	−2.5 (−2.8 to −2.2)	−1.5 (−1.8 to −1.2)
Mortality	24 685 (11.2)	121 749 (11.3)	57 625 (10.8)	183 054 (11.3)	−0.4 (−0.7 to −0.2)	−0.3 (−0.6 to −0.1)
SNF LOS (among 604 607 SNF stays), mean (SD), d	39.6 (35.0)	21.5 (21.8)	35.5 (32.5)	26.2 (25.6)	−8.3 (−8.8 to −7.7)	−6.3 (−6.8 to −5.8)
Improvement in ADL (among 473 043 SNF stays with assessments)	7097 (17.4)	26 799 (22.1)	18 825 (18.7)	41 753 (19.8)	3.9 (3.3 to 4.6)	2.7 (2.1 to 3.4)
Home health LOS (among 471 083 home health stays), mean (SD), d	35.7 (28.4)	33.0 (25.2)	33.6 (27.5)	36.6 (27.7)	−4.9 (−5.6 to −4.1)	−3.6 (−4.3 to −2.9)
Improvement in ADL (among 470 464 stays with assessments)	21 512 (63.2)	109 717 (79.7)	54 678 (67.1)	170 300 (78.4)	4.0 (3.1 to 4.9)	2.5 (1.7 to 3.4)
Days in the community, mean (SD), d	80.2 (33.9)	89.3 (24.0)	82.1 (32.0)	88.4 (25.7)	2.7 (2.4 to 3.0)	1.9 (1.7 to 2.2)

Abbreviations: ADL, activities of daily living; HHA, home health agency; LOS, length of stay; MA, Medicare Advantage; NA, not applicable; SNF, skilled nursing facility; TM, traditional Medicare.

^a^ Average treatment effect for the treated estimates are from a linear difference-in-differences model, adjusted for variables from the propensity score model and including a fixed effect for county of residence.

^b^ Presented as linear probability.

### Postacute Care Use Outcomes

Medicare Advantage beneficiaries had a 1.5–percentage point lower probability of readmission (95% CI, −1.8 to −1.2 percentage points) on a basis of 24.5% compared with TM beneficiaries (Table 2). The MA group also had 1.9 more days (95% CI, 1.7-2.2 days) in the community in the 100 days after hospital discharge compared with the TM group. Among beneficiaries surviving the index hospitalization, those in the MA group had a 0.3–percentage point lower probability of mortality (95% CI, −0.6 to −0.1 percentage points) compared with the TM group.

### Postacute Care Functional Outcomes

In the subsample of SNF users with ADL data (n = 473 043), MA beneficiaries had a 2.7–percentage point higher probability of 30-day ADL improvement (95% CI, 2.1-3.4 percentage points) compared with TM beneficiaries on a basis of 18.7% prevalence of 30-day ADL improvement in the TM group in 2015 (Table 2). Among home health users with complete ADL data (n = 470 464), the estimated probability of 30-day ADL improvement was 2.5 percentage points higher (95% CI, 1.7-3.4 percentage points) in the MA group compared with the TM group on a basis of 67.1% prevalence of 30-day ADL improvement.

### Sensitivity Analysis

Results of the sensitivity analyses were consistent with the main analysis, supporting the robustness of our conclusions. Additionally, we confirmed that changes in hospitalization diagnoses between 2015 and 2021 were similar for MA and TM beneficiaries and that adjusting our models for diagnosis-related group codes did not alter the results (eTables 4-7 in Supplement 1).

## Discussion

The combination of high costs, geographic variation, and poor outcomes has made postacute care a focus for improving health care value. This cohort study suggests that among Medicare beneficiaries likely to switch from TM to MA under expanded plan availability (compliers), SNF and home health use may decrease without worsening short-term to medium-term outcomes. These findings are particularly important as postacute care costs and use are expected to rise owing to an aging population and shorter hospital stays.^40^

Our results align with prior studies suggesting that MA enrollment results in fewer days of postacute care.^24^ For example, a study of 3 major MA insurers found that MA beneficiaries used 56% fewer SNF days than TM enrollees.^22^ Another study on patients with hip fractures found that MA beneficiaries spent 5.1 fewer days in SNFs compared with TM beneficiaries.^13^ Our finding that MA and TM beneficiaries used similar types of postacute settings contrasts with our initial hypothesis that MA would shift care from SNFs to home health services. A qualitative study has provided context, reporting that MA plans did not influence initial posthospital discharge settings (eg, SNF, home health, or home without postacute care).^11^ Similarly, hospital and SNF interviewees did not observe MA staff steering patients toward specific postacute care options.

Taken together, prior work and our findings suggest that SNF and home health care may be overused among some TM beneficiaries. However, it is unclear which mechanisms used in MA explain this difference. While tools such as prior authorization,^41^ cost-sharing,^42,43^ and reductions in SNF networks^44^ within MA have been identified as effective in reducing postacute care use, MA-like strategies carry potential downsides. While MA’s approach controls costs, hospital staff have reported that MA involvement often delays patient placement, impacting hospital length of stay.^43^ Narrow postacute care networks^44^ may limit patient choice. Claim denials might disincentivize facilities from admitting MA patients.^8^ Additionally, reductions in paid postacute care services under MA may shift care burdens from paid care to unpaid care provided by family caregivers, increasing the burden on informal caregivers.^45^

In addition, SNF and home health use declined in both MA and TM during our study period, suggesting that factors beyond MA payment incentives may contribute to this trend. We also did not assess any patient-reported outcomes, which are important to understand.

From a policy perspective, these results underscore the importance of existing opportunities to better align TM incentives with those in MA. Prior studies evaluating bundled payments for care improvement^36,46,47^ and Accountable Care Organizations^41,42^ have shown reductions in postacute care use in TM populations without a commensurate increase in harm. While the voluntary nature of bundled payments for care improvement may limit their broader impact, newer models such as the Transforming Episode Accountability Model, a mandatory program similar to the Comprehensive Care for Joint Replacement model, may help extend these gains to a broader TM population. Similarly, the Medicare Spending Per Beneficiary metric within hospital value-based purchasing could be leveraged to curb unnecessary postacute care. These examples suggest that well-designed payment models and accountability measures may promote efficient postacute care use in TM without relying on managed care structures.

### Strengths and Limitations

Our study had several strengths. It has built on prior research by using a comprehensive dataset covering 100% of the Medicare population and using robust methods to identify health care use and outcomes. By combining propensity score matching with difference-in-differences, we better controlled for unmeasured differences between MA and TM beneficiaries. We also incorporated county-level variables, such as MA plan availability and quality, to account for regional differences in MA enrollment. Extending the follow-up period to 100 days after discharge, rather than the typical 30-day window, enabled us to capture potential unintended consequences, such as long-term cost-shifting to Medicaid for nursing home care.

Our study also had several important limitations. First, we excluded beneficiaries with recent hospitalizations, SNF use, or home health use, as well as those with very long index hospital stays, to ensure comparability between the MA and TM groups. As a result, our findings may not be generalizable to high-need, high-cost patients who may experience adverse outcomes from changes in postacute care use. Second, without outpatient data, emergency department visits followed by discharge to home without home health care may have been missed, potentially resulting in misclassification of SNF days. Third, we did not account for the heterogeneity of plan types among MA beneficiaries due to a lack of details regarding MA plans. Inclusion of these data may help make the results more applicable to the current postacute care environment. Fourth, because of data limitations, we were unable to include discharges to inpatient rehabilitation facilities, an important setting to examine in this context. Finally, our ability to account for factors such as the role of caregivers was limited, leaving out key dynamics influencing care decisions and outcomes.

## Conclusions

This cohort study suggests that aligning TM incentives with MA strategies may enhance postacute care efficiency without compromising outcomes. Payment models such as bundled payments and Accountable Care Organizations offer TM-similar cost-saving potential. Policymakers must balance cost reduction with access and quality, ensuring that patient-reported outcomes and caregiver burdens are addressed in future reforms.

## Notes

Supplement 1.
eAppendix.
**eFigure 1.** Cohort Derivation Flowchart **eTable 1.** Comparison of Medicare Beneficiaries in 2015 and 2021 by Medicare Plan **eFigure 2.** Flowchart Describing Steps for Cross-Temporal Matching **eTable 2.** Comparison of Characteristics by Matched Groups **eFigure 3.** Covariate Balance Before and After Matching for the Creation of the Treatment and Control Groups **eTable 3.** Characteristics of Matched MA, TM, and Unmatched Groups and Absolute Standardized Mean Differences (SMDs) **eTable 4.** Observed Outcome Rates by Medicare Coverage Type Before Matching **eTable 5.** Observed Rates and Average Treatment Effect for the Treated (95% CI), MA 2015 to 2019 **eTable 6.** Distribution of the Top 10 DRG Codes Across 2015 and 2021 **eTable 7.** Estimation of Primary Models (Any SNF, Any HH, Readmission, and Mortality) With an Additional Adjustment for Hospitalization DRG Code

Supplement 2.
Data Sharing Statement
