# Supplementary material for: Postacute Care Use and Outcomes Among Medicare Advantage vs Traditional Medicare Beneficiaries
Source: JAMA Netw Open. 2025 Oct 29;8(10):e2540347. doi: 10.1001/jamanetworkopen.2025.40347 (PMC12573030; doi:10.1001/jamanetworkopen.2025.40347)
Supplement: Supplement 1. — eAppendix. eFigure 1. Cohort Derivation Flowchart eTable 1. Comparison of Medicare Beneficiaries in 2015 and 2021 by Medicare Plan eFigure 2. Flowchart Describing Steps for Cross-Temporal Matching eTable 2. Comparison of Characteristics by Matched Groups eFigure 3. Covariate Balance Before and After Matching for the Creation of the Treatment and Control Groups eTable 3. Characteristics of Matched MA, TM, and Unmatched Groups and Absolute Standardized Mean Differences (SMDs) eTable 4. Observed Outcome Rates by Medicare Coverage Type Before Matching eTable 5. Observed Rates and Average Treatment Effect for the Treated (95% CI), MA 2015 to 2019 eTable 6. Distribution of the Top 10 DRG Codes Across 2015 and 2021 eTable 7. Estimation of Primary Models (Any SNF, Any HH, Readmission, and Mortality) With an Additional Adjustment for Hospitalization DRG Code [file jamanetwopen-e2540347-s001.pdf]

Supplemental Online Content

Roy I, Huchins F, Rose L, et al. Postacute care use and outcomes among Medicare Advantage vs traditional Medicare beneficiaries. *JAMA Netw Open*. 2025;8(10):e2540347. doi:10.1001/jamanetworkopen.2025.40347

eAppendix.

eFigure 1. Cohort Derivation Flowchart

eTable 1. Comparison of Medicare Beneficiaries in 2015 and 2021 by Medicare Plan

eFigure 2. Flowchart Describing Steps for Cross-Temporal Matching

eTable 2. Comparison of Characteristics by Matched Groups

eFigure 3. Covariate Balance Before and After Matching for the Creation of the Treatment and Control Groups

eTable 3. Characteristics of Matched MA, TM, and Unmatched Groups and Absolute Standardized Mean Differences (SMDs)

eTable 4. Observed Outcome Rates by Medicare Coverage Type Before Matching

eTable 5. Observed Rates and Average Treatment Effect for the Treated (95% CI), MA 2015 to 2019

eTable 6. Distribution of the Top 10 DRG Codes Across 2015 and 2021

eTable 7. Estimation of Primary Models (Any SNF, Any HH, Readmission, and Mortality) With an Additional Adjustment for Hospitalization DRG Code

This supplemental material has been provided by the authors to give readers additional information about their work.

© 2025 Roy I et al. *JAMA Network Open*.

eAppendix

Overall Methodological Strategy

To address the persistent challenge of selection bias in comparing outcomes between Medicare Advantage (MA) and traditional Medicare (TM) enrollees, we take advantage of the substantial growth in MA enrollment between 2015 and 2021. Rather than relying on cross-sectional matching of MA enrollees in 2021 to similar TM beneficiaries in 2021, which may inadequately adjust for unobserved factors like health preferences or care-seeking behavior, we adopt a cross-temporal matching strategy. Specifically, we match MA enrollees from 2021 to TM beneficiaries from 2015 who are otherwise similar in terms of their propensity to enroll in MA, taking advantage of the differential availability and prevalence of MA in 2021 versus 2015. The “complier” group in this analysis is TM beneficiaries who would have enrolled in MA in 2015 (using factors in our propensity score most associated with MA enrollment) had it been as prevalent then as it was in 2021. This approach improves the likelihood of identifying TM “matches” who most closely resemble the types of individuals who enrolled in MA by 2021, thereby reducing bias due to changing enrollee composition over time.

Cross-sectional propensity score matching assumes unconfoundedness, meaning that after adjusting for observed characteristics, treatment assignment is independent of outcomes. In this setting, that assumption is tenuous because MA enrollment is influenced by factors not observed in claims data, such as preferences for managed care, provider trust, or informal caregiver availability. Our approach instead matches on the likelihood of MA enrollment, which reflects variation in MA availability across time and markets. By leveraging shifts in enrollment opportunities rather than relying only on outcome-related characteristics, the design provides stronger grounds for causal interpretation.

To account for secular trends in post-acute care use and outcomes that are unrelated to insurance type, we implement a difference-in-differences (DiD) framework. Standard DiD require that treatment and control groups would have followed parallel trends in the absence of treatment. This assumption is threatened in our setting because MA growth altered who enrolled over time. As shown in eTable 1, the composition of MA enrollees in 2015 differs from that in 2021, making traditional DiD comparisons problematic. Cross-temporal matching mitigates this concern by aligning comparable beneficiaries across periods, and DiD then isolates the effect of MA enrollment from broader temporal changes in healthcare practice, policy, or population health.

© 2025 Roy I et al. *JAMA Network Open*.

eFigure 1. *Cohort Derivation Flowchart*

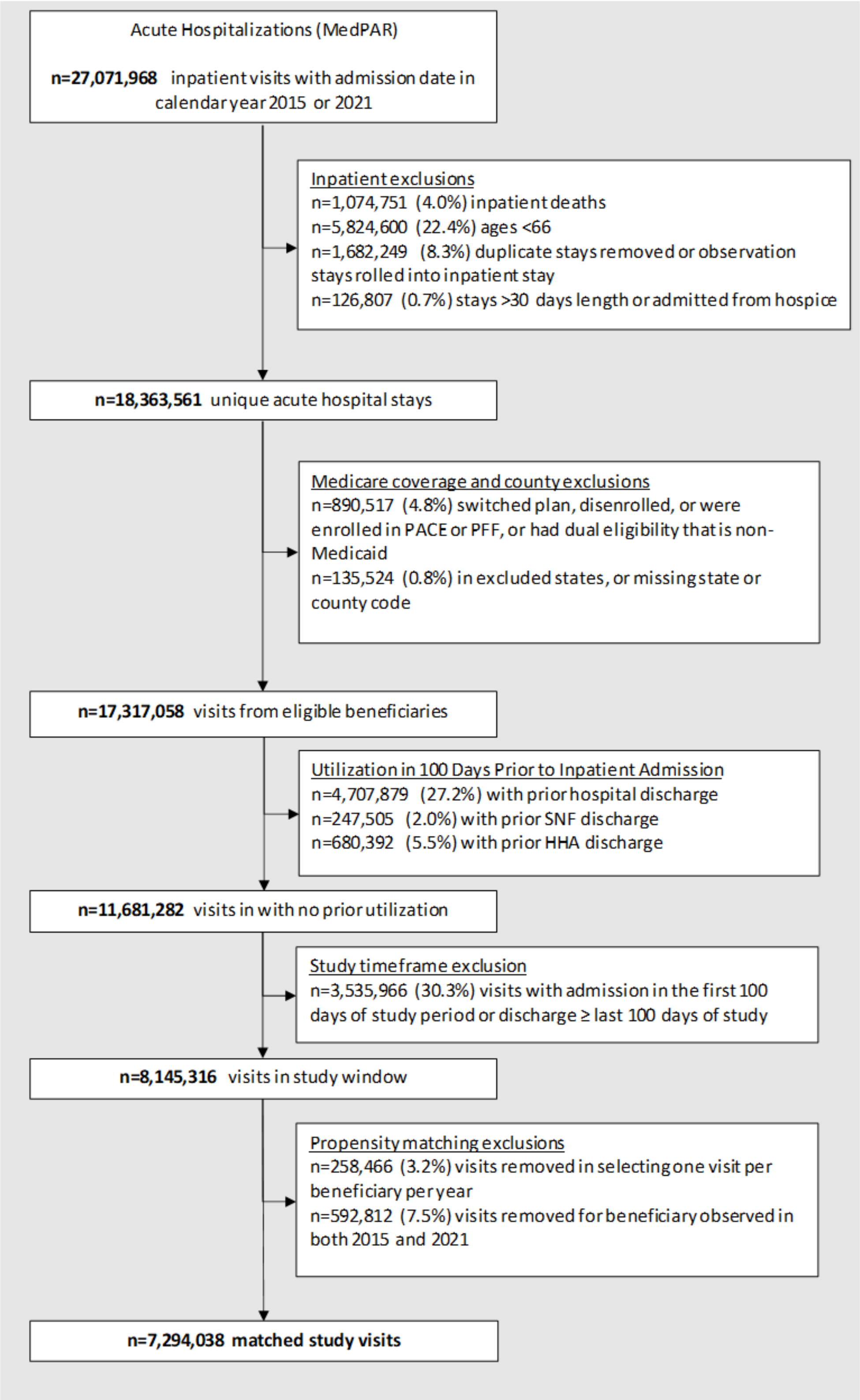

© 2025 Roy I et al. *JAMA Network Open*.

Implementation of the Cross-Temporal Propensity Score Matching Estimation Strategy

To clarify our matching strategy, recall that the study considers three potential groups of individuals.

Group 1 includes Medicare beneficiaries who were enrolled in MA in 2015 (pre-period) and are matched to MA enrollees in 2021 (post-period). This group represents the "always-takers" (or "always-MA"). Specifically:

1. **$G1_{2015}$**: MA beneficiaries observed in 2015 who chose MA even when plan availability was more limited.
2. **$G1_{2021}$**: MA beneficiaries observed in 2021 who are matched to $G1_{2015}$ and are likewise likely to always choose MA, regardless of availability.

Thus, members of Group 1 are exposed to MA in both years. Because it is not possible to estimate their outcomes under a scenario without MA exposure, they are excluded from the main analyses.

Group 2 is the primary group used to estimate the effect of MA enrollment on post-acute care use and outcomes (the "compliers", or *treatment group*). It includes individuals who:

1. Were not enrolled in MA in 2015 ($G2_{2015}$), and
2. Are propensity-matched to individuals who enrolled in MA in 2021 (after excluding those in Group 1, $G2_{2021}$).

These "new" MA users allow us to compare outcomes under different exposure states: the 2015 individuals represent outcomes under *no MA exposure*, while their 2021 matches represent outcomes *with MA exposure*.

Group 3 is the *control group (the "never takers")*. It consists of beneficiaries who were enrolled in TM in 2015 ($G3_{2015}$) and are propensity-matched to beneficiaries enrolled in TM in 2021 ($G3_{2021}$). This represents the group that chooses TM even when MA availability is high. Changes in outcomes for this group are important to capture to measure secular trends in post-acute care use and outcomes that occur over time for reasons unrelated to MA enrollment. These trends serve as a baseline against which changes observed in Group 2 can be compared (ie, as if Group 2 had remained in TM during this time).

Steps to implement the cross-temporal matching:

Step 1. Separation of post-period 2021 MA Users into $G1_{2021}$ (always MA) and $G2_{2021}$ (new MA) (see Figure 1 of the paper):
1.1. Estimate a propensity score for MA use with our 2015 baseline cohort, $\mathbf{p_{2015}}$. C statistic for logistic regression was 0.66.
1.2. Use the $\mathbf{p_{2015}}$ model to predict the propensity score for the 2021 cohort.
1.3. Use these propensity scores to find 1:1 matches with replacement for the 2015 MA enrollees among the 2021 MA enrollees ($G1_{2021}$ in Figure 1).
1.4. The 2021 MA enrollees left unmatched when creating Group 1 become our predicted "new MA" users in Group 2.

Step 2. Construction of Group 2 (treatment group or MA):
2.1. Estimate a propensity score with the 2021 post-baseline cohort, $\mathbf{p_{2021}}$. C-statistic for logistic regression model was 0.72.

© 2025 Roy I et al. *JAMA Network Open*.

2.2. Use the $\mathbf{p_{2021}}$ model to predict the propensity score for the remaining beneficiaries in the 2015 cohort.
2.3. Use these propensity scores to find 1:1 matches (with replacement) for the individuals in $G2_{2021}$ among 2015 TM enrollees ($G2_{2015}$ in Figure 1).

Step 3. Construction of Group 3 (Control group or TM):
3.1. Use $\mathbf{p_{2021}}$ to find 1:1 matches (with replacement) for the post-baseline 2021 TM beneficiaries ($G3_{2021}$) among 2015 TM beneficiaries ($G3_{2015}$)

The 1:1 matching was done using the nearest neighbor (closest) propensity score method, and it was done with replacement to allow the selection of the closest propensity score for each person to be done from the entire pool of potential matching candidates. Weights were then used to account for a candidate being selected multiple times. Because the matching is done with replacement, there were 2015 TM beneficiaries that were not selected into the $G2_{2015}$ or $G3_{2015}$ groups as best matches by any of the 2021 beneficiaries. They are labelled as unmatched (UNM_{year}) in eTable 2.

Groups 2 and 3 are the two groups used to carry out the DID analysis using the following regression model:

$$Y = a_0 + a_1 TRT + a_2 Post + \delta TRT \times Post + bX + \epsilon$$

where TRT is a 0/1 indicator of whether the individual belonged to the MA group ($G2_{2015}$ or $G2_{2021}$), *Post* is a 0/1 indicator of the observation corresponding to the post-baseline year 2021, X is the list of covariates used in the propensity models and county fixed effects to adjust for residual imbalance in our matched Groups 2 and 3, and ϵ is the error term. The desired MA enrollment-outcome relationship is given by the coefficient of the interaction term δ, which estimates the changes in outcomes over time for Group 2 minus the changes in outcomes over time for Group 3, ($G2_{2021}$-$G2_{2015}$) – ($G3_{2021}$- $G3_{2015}$).

Rationale for Covariate Selection

We selected covariates based on their potential to confound the relationship between Medicare Advantage (MA) enrollment and post-acute care outcomes. Consistent with prior studies, we included characteristics known to predict MA enrollment and factors that may also influence outcomes.

First, we accounted for demographic characteristics—age, sex, and race/ethnicity—which have been shown to shape beneficiaries' choice of MA versus Traditional Medicare (TM). To capture socioeconomic status, we included dual eligibility as an indicator of insurance coverage and financial need, as well as the Area Deprivation Index as a neighborhood-level measure of socioeconomic disadvantage. Second, because MA availability is strongly geographically determined, we adjusted for county-level market characteristics. These included the number and quality of MA plans available, the size of the Medicare population, benchmark payment rates (which influence plan entry and generosity), and an indicator for rural versus urban residence. Together, these variables help account for local market dynamics that affect both enrollment decisions and access to care. We also accounted for availability of hospitals, SNF and HH facilities at the county level. Third, to reflect beneficiaries' underlying health status prior to hospitalization, we included the Charlson comorbidity index, a claims-based frailty measure, and the number of prior hospitalizations. These measures capture variation in medical complexity and

© 2025 Roy I et al. *JAMA Network Open*.

functional risk that influence both treatment choice and subsequent care needs. We also included the original reason for Medicare entitlement (age, disability, or ESRD), given its established association with MA enrollment.

Importantly, we did not adjust for characteristics of the index hospitalization, such as diagnosis or length of stay. These factors occur after the enrollment decision and are therefore downstream of treatment assignment. Including such post-treatment variables could induce bias by conditioning on consequences of MA enrollment, rather than confounders measured at or before the time of treatment assignment.

Quality of Propensity Score Matching

We assessed the quality of the propensity score matching in two steps. First, we checked to see whether the factors associated with the MA enrollment propensity scores changed in 2015 compared to the 2021 cohort and verified that the propensity scores were balanced across MA and TM subgroups for each of our three major groups (Group 1, Group 2 and Group 3). Second, we verified that the covariates were properly balanced across pre- and post- period groups (eTable 2, eFigure 3a & b).

The logistic regression models used to construct the two propensity scores, p2015 and p2021, showed that estimated odds ratios for several predictors shifted substantially between 2015 and 2021. Based on these results, we used the 2015 propensity score model to classify 2021 MA enrollees into two groups: those who would likely have enrolled in MA even in 2015 (Group 1, “always-takers”) and those who would likely have remained in TM in 2015 but enrolled in MA by 2021 as availability increased (Group 2, “compliers”). The c-statistics (area under the ROC curve) were 0.66 and 0.72 respectively, indicating adequate sensitivity and specificity.

The most critical validation is to verify that after the propensity score matching the covariates are properly balanced within each of the three groups. eTable 2 provides the averages of the covariates for each group in the first seven columns, while the last three columns display the absolute standardized differences, defined as the absolute difference of the sample means in the treated (original subgroup) and non-treated (matched subgroup) subsamples of a given group, standardized by the square root of the average of the sample variances in the treated and non-treated sub-samples of a given group. eFigure 3a and 3b show the covariate balance plot. There is no agreement criterion as to what threshold of the standardized difference should be used to indicate imbalance. Some studies have used a standard difference less than 0.1 to indicate a negligible difference in the mean or prevalence of a covariate between different treatment groups. In our case, groups had negligible difference on Charlson comorbidity index, frailty score, prior hospital use, with standard difference less than 0.1.

Residual imbalance after matching, such as that observed in the county level variables such as MA plan availability or in the race/ethnicity variables was controlled by including all the covariates in DiD regression model. Models accounted for clustering at the counties to produce doubly adjusted robust estimates.

© 2025 Roy I et al. *JAMA Network Open*.

Identifying assumptions of the overall approach:

Assumption 1 The stable unit treatment value assumption (SUTVA)

In this study, the Stable Unit Treatment Value Assumption (SUTVA) is assumed to hold. SUTVA requires that (1) there is no interference between units, meaning the treatment status of one beneficiary does not affect the outcome of another, and (2) there is only one version of the treatment. In our context, Medicare Advantage (MA) enrollment is well-defined and consistently regulated across beneficiaries, and an individual's post-acute care use or outcomes are unlikely to be directly influenced by whether other beneficiaries are enrolled in MA or traditional Medicare (TM). Regarding the second component, while there is heterogeneity across MA plans, all plans operate under fundamentally the same regulatory and financial incentives. Although individual plans may respond differently to these incentives, the exposure we are measuring, which is the beneficiary's response to the standardized set of MA incentives, remains conceptually uniform. Thus, although plan-level variation exists, the treatment can be reasonably considered as a single, well-defined exposure. Furthermore, while MA penetration has grown substantially over time, our difference-in-differences (DiD) framework accounts for secular trends that may reflect system-level effects of this growth.

Assumption 2: Unconfounded encouragement

The "encouragement" in our natural experiment is the increased availability and uptake of MA plans between 2015 and 2021. This encouragement is assumed to be related to MA enrollment in an equivalent way across units. Prior literature shows that the propensity to enroll in MA can be predicted using observable beneficiary and market characteristics, suggesting enrollees respond to MA plan availability and generosity in similar ways (Park S, et al., 2023; Atherly A, et al., 2023).

We also assume that the assignment of the encouragement is unconfounded with both potential intervention exposure and potential outcomes, conditional on observed covariates. All individuals in our analytic sample had a non-zero probability of experiencing the encouragement (positivity assumption). To ensure this, we excluded beneficiaries in counties without any MA plan availability, ensuring that all eligible individuals had some opportunity to enroll in MA. Furthermore, MA and TM hospitalizations are observed equivalently in both time periods, and observation of hospitalization is not dependent on subsequent PAC use.

Assumption 3: The strong monotonicity assumption

This assumption requires that beneficiaries who would enroll in MA in 2015 would also enroll in 2021 (i.e., no defiers). While some beneficiaries can move from MA back to TM (defiers), this is not the overall population trend. In addition, there is at least 1 individual who was not exposed to MA in 2015, but would be exposed in 2021.Under this assumption, beneficiaries can be classified into three groups: always-takers, who would enroll in MA in both 2015 and 2021; never-takers, who would not enroll in MA in either year; and compliers, who were not enrolled in 2015 but enrolled in 2021. This assumption is reasonable for our study given the substantial national growth in MA penetration between 2015 and 2021.

© 2025 Roy I et al. *JAMA Network Open*.

Traditionally, in instrumental variable analyses, monotonicity is understood as a linear and positively correlated relationship between the instrument and the treatment. For example, the closer a patient lives to a particular facility, the more likely they are to choose it. In our longitudinal setting, we interpret time as the instrument, assuming that the trend over time increases the likelihood of MA enrollment in a similarly monotonic way across beneficiaries. This interpretation ensures that treatment assignment over time is predictable and that defiers are negligible, supporting the plausibility of the assumption for our study.

Assumption 4: The stratum-specific common temporal trends assumption

This assumption says that, within compliance strata, the average temporal effects are the same for units with the same exposure status. In our study, the strata are always-MA (enrolled in MA in both 2015 and 2021), always-TM (enrolled in traditional Medicare in both years), and compliers (transitioning from TM in 2015 to MA in 2021). The assumption implies that, in the absence of changes in MA enrollment, the average change in outcomes for compliers would match that of always-TM beneficiaries. In other words, if MA availability had remained constant, outcomes for the MA growth group would have followed the same temporal trend as those always in TM.

Standard difference-in-differences analyses rely on a global parallel trends assumption, which may be violated due to heterogeneity across strata. For example, always-MA beneficiaries may experience different temporal changes than always-TM beneficiaries because of differences in health status, utilization, or local market factors. By using a stratum-specific common temporal trends assumption, we allow trends to vary across strata while still isolating the causal effect of MA enrollment growth, extending the parallel trends concept in a more flexible and plausible way.

eTable 1. *Comparison of Medicare Beneficiaries in 2015 and 2021 by Medicare Plan*

Covariate	2015<br>N= 3704118			2021<br>N= 3589920		
N (%)	TM<br>N=<br>2,569,280<br>(69.4%)	MA<br>N=<br>1,134,838<br>(30.6%)	Total	TM<br>N=<br>2,037,749<br>(56.8%)	MA<br>N=<br>1,552,171<br>(43.2%)	Total
Age (Mean, std)	78.39 (8.32)	77.74 (7.86)	78.19 (8.18)	77.95 (8.17)	77.38 (7.82)	77.70 (8.02)
Male (%)	44.09	44.39	44.18	46.70	45.88	46.34
Race/Ethnicity						
American Indian	0.50	0.20	0.41	0.57	0.26	0.44
Asian / Pacific Islander	1.86	2.44	2.04	2.23	2.85	2.50
Hispanic	4.56	9.57	6.09	4.89	10.80	7.45
Non-Hispanic Black	7.94	11.11	8.91	7.56	13.49	10.12

© 2025 Roy I et al. *JAMA Network Open*.

Non-Hispanic White	83.94	75.49	81.35	82.49	70.74	77.41
Other / Unknown	1.20	1.19	1.20	2.26	1.86	2.09
Dual-Eligibility	17.50	19.24	18.03	15.32	23.90	19.03
Weighted Charlson Comorbidity Index, mean (std)	2.21 (2.18)	2.34 (2.25)	2.25 (2.20)	2.61 (2.45)	2.83 (2.50)	2.70 (2.47)
Frailty Score, mean (std)	0.18 (0.04)	0.17 (0.04)	0.17 (0.04)	0.18 (0.04)	0.18 (0.04)	0.18 (0.04)
Area Deprivation Index, mean (std)	51.81 (21.25)	50.53 (20.42)	51.42 (21.01)	50.83 (21.58)	51.09 (21.31)	50.94 (21.46)
Medicare Population in Beneficiary County, mean (std)	104747.49 (137788.35)	133039.52 (139500.69)	113401.48 (138927.34)	153521.42 (250665.16)	193533.19 (287868.88)	170894.25 (268189.42)
Availability of MA plans in Beneficiary County, mean (std)	15.40 (11.06)	20.60 (11.10)	17.00 (11.33)	62.24 (29.93)	70.92 (28.47)	66.03 (29.62)
Benchmark rate, mean (std)	774.94 (53.50)	779.42 (68.41)	776.31 (58.51)	984.49 (45.97)	979.62 (44.69)	982.39 (45.49)
MA Plan Rating in Beneficiary County, mean(std)	3.79 (0.02)	3.79 (0.03)	3.79 (0.02)	3.91 (0.02)	3.91 (0.02)	3.91 (0.02)
Hospitals per 1000 Beneficiary in County,	0.26 (0.32)	0.25 (0.33)	0.26 (0.33)	0.20 (0.23)	0.18 (0.16)	0.19 (0.20)

© 2025 Roy I et al. *JAMA Network Open*.

mean (std)						
SNF/HHA per 1000 Beneficiary in County, mean (std)	1.42 (1.91)	1.53 (2.43)	1.45 (2.09)	0.88 (0.64)	0.86 (0.58)	0.87 (0.61)
Rural Residence	19.67	11.10	17.05	18.82	12.82	16.23
Original Medicare Eligibility due to Age	87.00	85.49	86.54	85.96	80.98	83.81
Prior Hospitalizations in the year before index acute stay						
None	78.12	80.39	78.82	81.70	83.16	82.33
1	14.92	13.78	14.57	12.65	11.86	12.31
2	4.45	3.82	4.26	3.61	3.25	3.46
3 or more	2.50	2.00	2.35	2.04	1.73	1.91
SOURCE [Author's analysis of data from the Medicare Provider Analysis and Review (MedPAR), Minimum Data Set (MDS) 3.0, Outcome and Assessment Information Set (OASIS), and publicly available CMS data files on MA enrollment and plans 2015-2021]						

© 2025 Roy I et al. *JAMA Network Open*.

eFigure 2. *Flowchart Describing Steps for Cross-Temporal Matching*

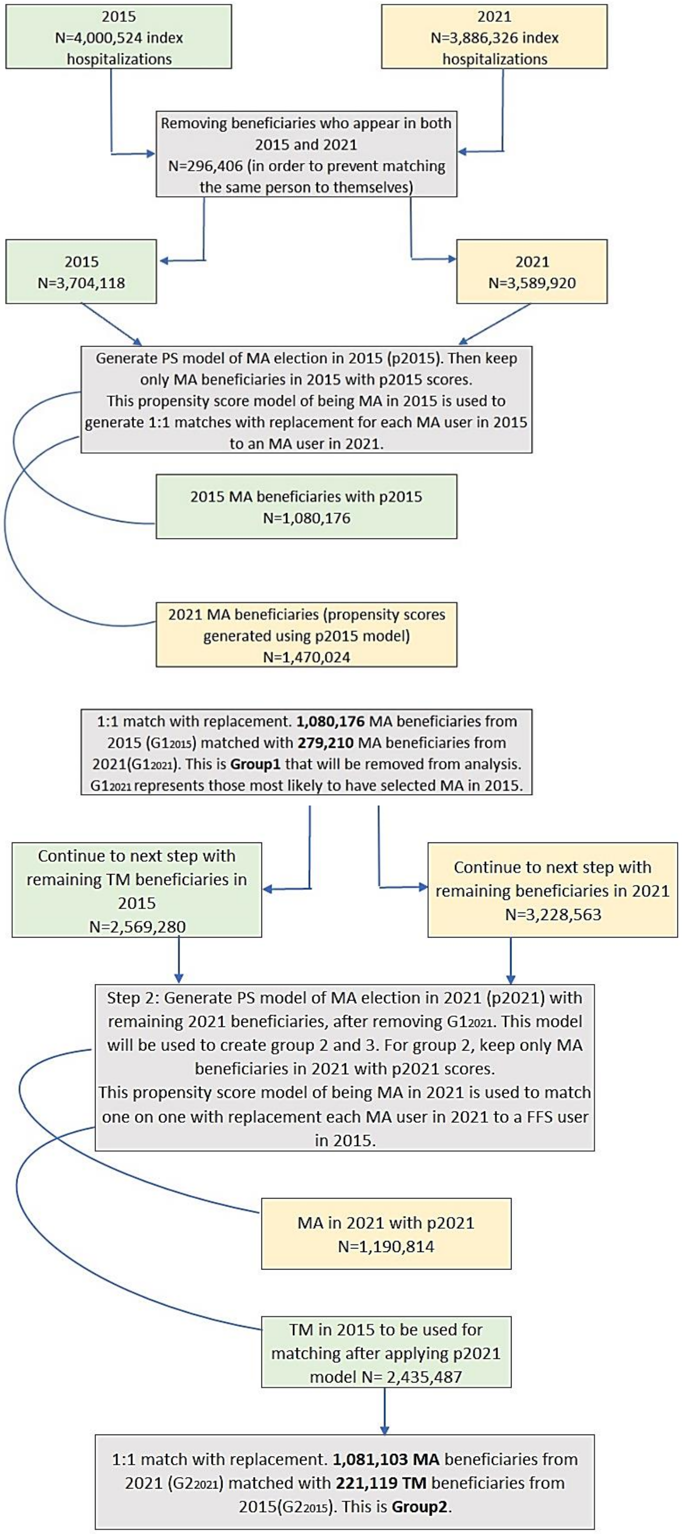

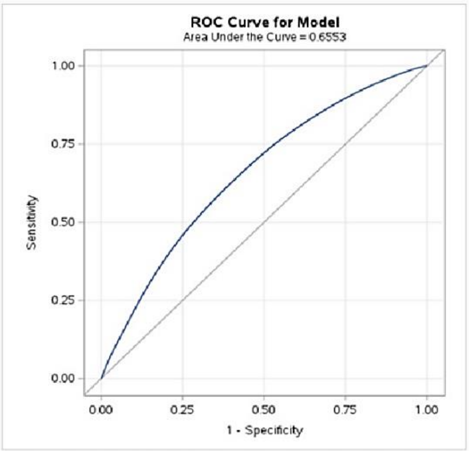

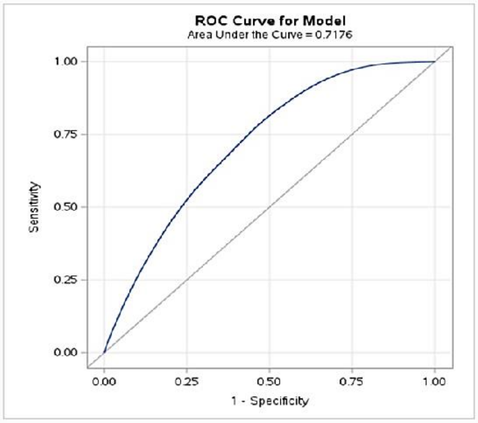

© 2025 Roy I et al. *JAMA Network Open*.

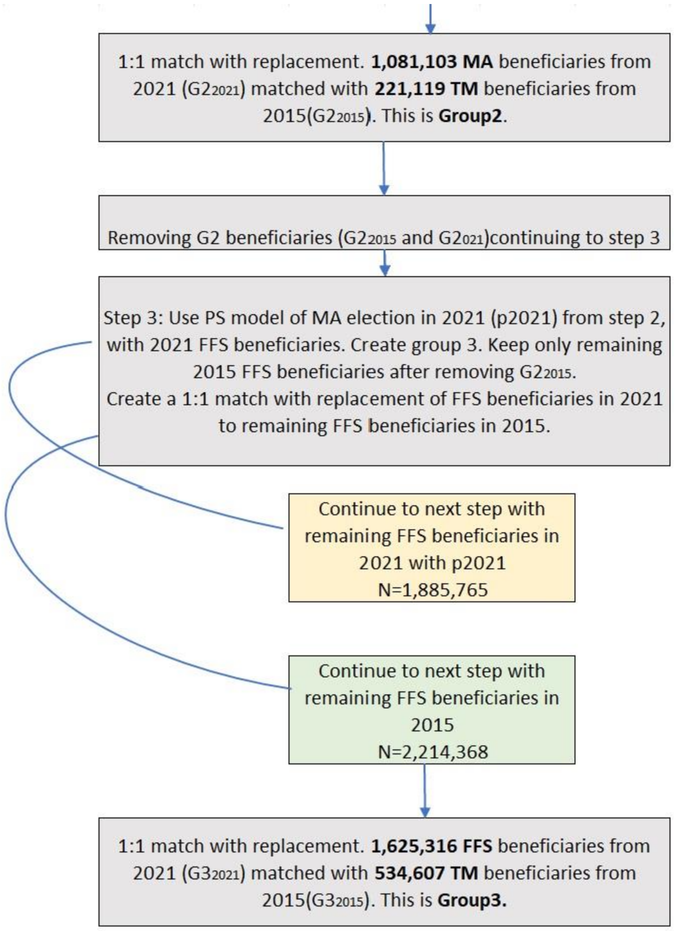

© 2025 Roy I et al. *JAMA Network Open*.

eTable 2. Comparison of Characteristics by Matched Groups

	**2015**				**2021**					**Absolute Standardized Differences**		
Group	**$G1_{2015}$**	**$G2_{2015}$**	**$G3_{2015}$**	**UM_{2015}**	**$G1_{2021}$**	**$G2_{2021}$**	**$G3_{2021}$**	**UM_{2021}**	**UM_{2021}**	**$G1_{2015}$ vs $G1_{2021}$**	**$G2_{2015}$ vs $G2_{2021}$**	**$G3_{2015}$ vs $G3_{2021}$**
Plan	MA	TM	TM	TM	MA	MA	TM	MA	TM			
N (unique hospitalizations)	1,080,176	2,435,487	2,214,368	1,679,761	1,470,024	1190814	1,885,765	109,711	260,449			
N (weighted for matching)	1,080,176	221,119	534,607		279,210	1,081,103	1,625,316					
Age	77.75	76.29	78.07	78.80	77.81	77.52	78.20	75.15	76.45	0.008	0.157	0.015
Male (%)	44.31	42.19	43.85	44.37	46.02	46.10	46.87	42.92	45.24	0.034	0.079	0.061
Race/Ethnicity *(%)*												
American Indian	0.21	0.04	0.37	0.62	0.71	0.18	0.65	0.00	0.01	0.075	0.043	0.039
Asian / Pacific Islander	2.53	2.55	2.39	1.68	2.11	3.37	1.86	0.95	5.27	0.028	0.049	0.037
Hispanic	8.61	25.17	5.20	1.43	3.53	9.28	2.23	48.86	22.74	0.214	0.431	0.157
Non-Hispanic Black	10.90	34.06	10.03	3.55	8.01	12.85	4.55	33.75	25.58	0.099	0.517	0.212
Non-Hispanic White	76.55	37.73	80.92	91.37	83.78	72.28	88.26	16.13	45.15	0.182	0.741	0.204
Other / Unknown	1.21	0.45	1.10	1.35	1.87	2.03	2.45	0.30	1.26	0.053	0.144	0.103
Dual-Eligibility (%)	18.76	43.64	19.82	13.09	20.91	20.92	12.03	59.12	35.86	0.054	0.501	0.214
Weighted Charlson Comorbidity Index	2.35	2.88	2.31	2.08	2.71	2.79	2.50	3.64	3.24	0.155	0.038	0.081

© 2025 Roy I et al. *JAMA Network Open*.

Frailty Score	0.17	0.18	0.18	0.18	0.18	0.18	0.18	0.18	0.18	0.158	0.036	0.004
Area Deprivation Index	50.33	56.86	52.79	50.56	56.35	49.36	50.50	50.26	50.69	0.274	0.380	0.108
Medicare Population in Beneficiary County	133875.79	131903.23	108037.63	101041.44	54085.51	216528.32	136211.28	368933.68	290696.06	0.721	0.363	0.151
Availability of MA plans in Beneficiary County	20.50	21.15	17.00	14.38	35.25	77.62	57.13	105.05	99.10	1.245	3.118	1.941
Benchmark rate	774.11	764.03	770.56	776.66	987.13	979.44	987.06	973.09	973.47	4.113	5.118	4.603
MA Plan Rating in Beneficiary County	3.79	3.80	3.80	3.79	3.91	3.91	3.91	3.91	3.91	5.516	4.769	5.124
Hospitals per 1000 Beneficiary in County	0.25	0.19	0.24	0.28	0.24	0.16	0.20	0.15	0.15	0.041	0.185	0.131
SNF/HHA per 1000 Beneficiary in County	1.53	1.04	1.29	1.52	0.94	0.84	0.87	0.89	0.88	0.331	0.305	0.338
Rural Residence (%)	10.66	3.08	14.44	22.48	36.44	6.94	20.21	0.10	0.50	0.638	0.177	0.153
Original Medicare Eligibility due to Age (%)	85.50	70.23	85.07	89.89	81.26	82.92	87.83	62.22	74.28	0.114	0.303	0.081
Prior hospitalization (%)												
None	80.41	83.17	79.61	77.00	81.52	83.13	81.24	87.48	84.23	0.028	0.001	0.041
1	13.77	12.23	14.51	15.52	12.73	11.89	12.91	9.44	11.17	0.031	0.010	0.036

© 2025 Roy I et al. *JAMA Network Open*.

2	3.82	3.15	4.04	4.75	3.67	3.25	3.72	2.19	3.02	0.008	0.006	0.016
3 or more	1.99	1.44	2.21	2.73	2.08	1.73	2.13	0.89	1.57	0.006	0.023	0.005
UM refers to observations that were left unmatched												

© 2025 Roy I et al. *JAMA Network Open*.

__eFigure 3.__ Covariate Balance Before and After Matching for Creation of the Treatment and Control Groups

eFigure 3a: Figure shows covariate balance before and after matching for creation of treatment group.

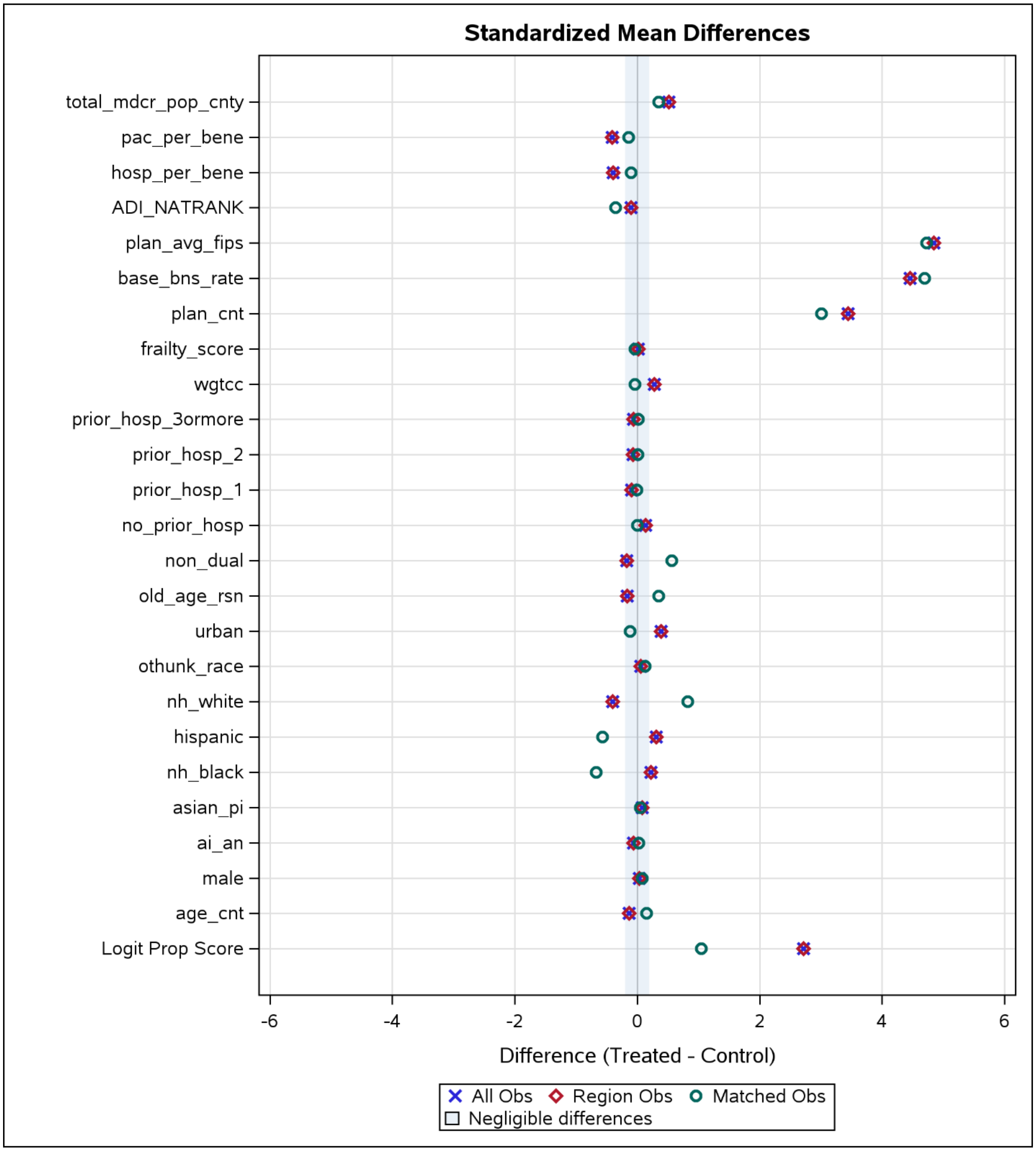

© 2025 Roy I et al. *JAMA Network Open*.

eFigure 3b: Figure shows covariate balance before and after matching for creation of control group.

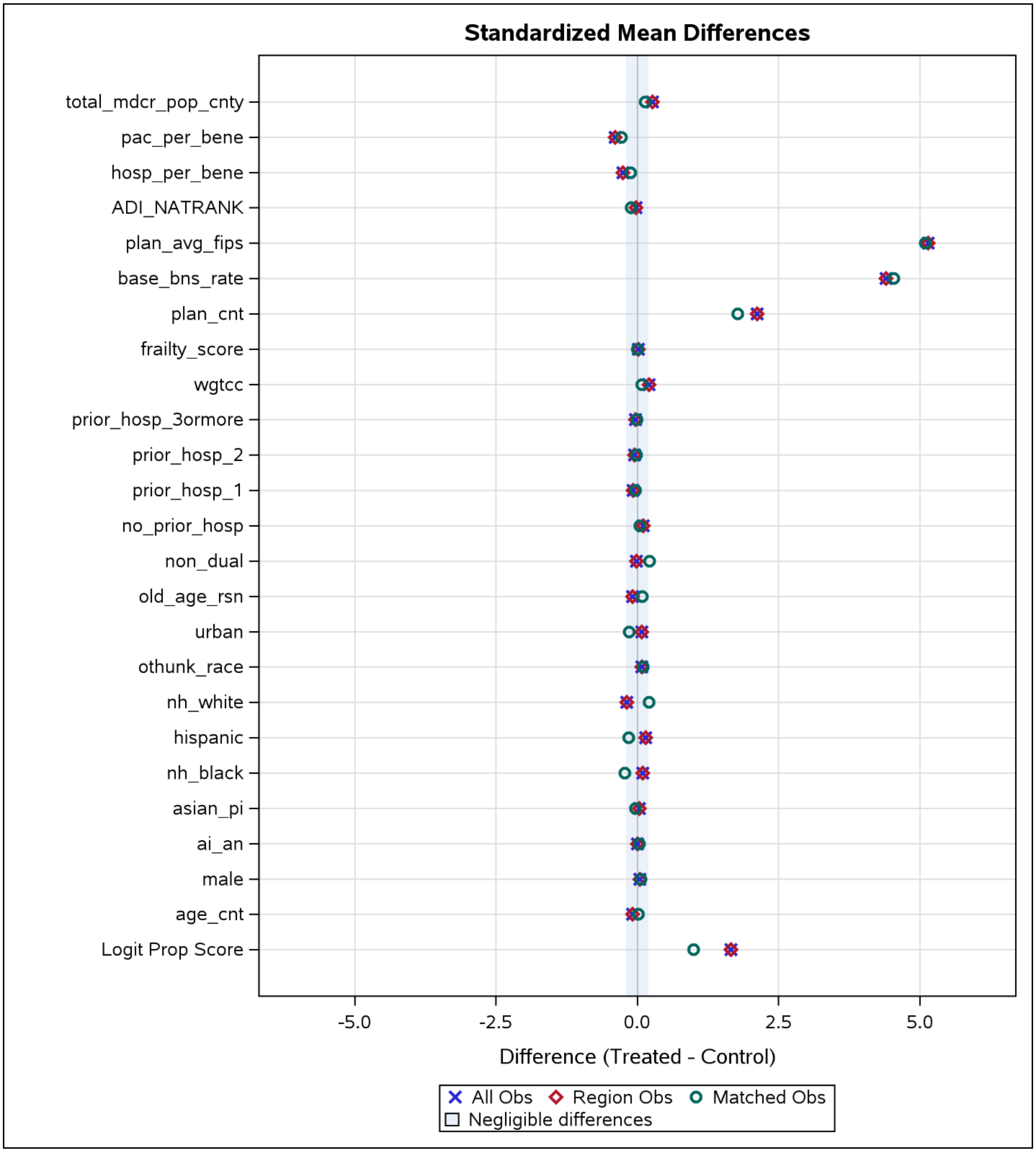

Variable index displayed in eFigure 3a and 3b	
**Total_mdcr_pop_cnty**	**Total Medicare population at beneficiary's county of residence**

© 2025 Roy I et al. *JAMA Network Open*.

**Pac_per_bene**	**Number of SNF +HH facilities available per 1000 beneficiary at county of residence**
**Hosp_per bene**	**Number of hospitals available per 1000 beneficiary at county of residence**
**ADI_Natrank**	**Area Deprivation Index measure**
**Plan_avg_fips**	**Average rating of MA plans at county of residence**
**Base_bns_rate**	**MA plan benchmark payment rate at the county**
**Plan_cnt**	**Number of MA plans available at county of residence**
**Frailty_score**	**Kim's frailty score**
**wgtcc**	**Weighted Charlson Score**
**Prior_hosp_3ormore**	**More than 3 prior hospitalizations in the year before index hospitalization**
**Prior_hosp_2**	**Two hospitalizations in the year before index hospitalization**
**Prior_hosp_1**	**One hospitalization in the year before index hospitalization**
**No_prior_hosp**	**No prior hospitalization in the year before index hospitalization**
**Non_dual**	**Medicare only**
**Old_age_rsn**	**Enrollment in Medicare due to old age**
**urban**	**Beneficiary resides in urban area**
**Othunk_race**	**Beneficiary race other or unknown**
**Nh_white**	**Beneficiary Non-Hispanic White**
**Hispanic**	**Beneficiary Hispanic**
**NH_Black**	**Beneficiary Black**
**Asian_pi**	**Beneficiary Asian/Pacific Islander**
**Ai_an**	**Beneficiary American Indian/Alaska Native**
**Male**	**Beneficiary male**
**Age_cnt**	**Beneficiary age as continuous**

© 2025 Roy I et al. *JAMA Network Open*.

eTable 3. *Characteristics Matched MA, TM, and Unmatched Group and Absolute Standardized Mean Differences (SMDs)*

N (%)	Unmatched<br><br>N=1,940,210	MA<br>N=1,302,222	TM<br>N=2,159,923	Absolute SMD (MA vs TM)	Absolute SMD (unmatched vs MA)	Absolute SMD (unmatched vs TM)
Age (Mean, std)	78.48 (8.34)	77.32 (7.86)	78.17 (8.20)	0.1066	0.1446	0.0382
Male	44.49	45.44	46.12	0.0136	0.0191	0.0327
Race/Ethnicity						
American Indian	0.54	0.16	0.58	0.0704	0.0650	0.0060
Asian / Pacific Islander	2.17	3.23	1.99	0.0780	0.0658	0.0124
Hispanic	4.29	11.98	2.97	0.3479	0.2841	0.0708
Non-Hispanic Black	6.50	16.45	5.91	0.3395	0.3160	0.0248
Non-Hispanic White	85.16	66.41	86.44	0.4855	0.4485	0.0367
Other / Unknown	1.34	1.76	2.11	0.0254	0.0342	0.0593
Dual-Eligibility	16.15	24.77	13.96	0.2763	0.2151	0.0612
Weighted Charlson Comorbidity Index, mean (sd)	2.23 (2.22)	2.80 (2.49)	2.45 (2.35)	0.1439	0.2405	0.0956
Frailty Score, mean (sd)	0.18 (0.04)	0.18 (0.04)	0.18 (0.04)	0.0166	0.0198	0.0032
Area Deprivation Index, mean (sd)	50.58 (21.58)	50.63 (20.55)	51.07 (21.58)	0.0206	0.0027	0.0227
Medicare Population in Beneficiary County, mean (sd)	126500.21 (196896.54)	202158.87 (280639.45)	129237.96 (208402.37)	0.2950	0.3121	0.0135
Availability of MA plans in Beneficiary County, mean (sd)	25.76 (31.54)	68.03 (25.76)	47.20 (29.70)	0.6951	1.368	0.7000

© 2025 Roy I et al. *JAMA Network Open*.

Benchmark rate, mean (sd)	803.08 (83.01)	942.86 (91.46)	933.48 (104.48)	0.0956	1.601	1.382
MA Plan Rating in Beneficiary County, mean(sd)	3.81 (0.04)	3.89 (0.05)	3.88 (0.05)	0.1663	1.748	1.468
Hospitals per 1000 Beneficiary in County, mean (sd)	0.26 (0.33)	0.17 (0.13)	0.21 (0.25)	0.2114	0.3668	0.1698
SNF/HHA per 1000 Beneficiary in County, mean (sd)	1.43 (1.98)	0.87 (1.43)	0.98 (1.01)	0.1275	0.3832	0.2898
Rural Residence	19.53	6.28	18.78	0.3845	0.4031	0.0190
Original Medicare Eligibility due to Age	87.80	80.77	87.15	0.1745	0.1940	0.0196
Prior Hospitalizations in the year before index acute stay						
None	77.97	83.13	80.83	0.0599	0.1307	0.0708
1	14.94	11.95	13.21	0.0381	0.0876	0.0495
2	4.52	3.23	3.80	0.0308	0.0666	0.0360
3 or more	2.57	1.68	2.15	0.0342	0.0617	0.0277
SOURCE [Author’s analysis of data from the Medicare Provider Analysis and Review (MedPAR), Minimum Data Set (MDS) 3.0, Outcome and Assessment Information Set (OASIS), and publicly available CMS data files on MA enrollment and plans 2015-2021] Table compares characteristics of 1,940,210 beneficiaries who were not included in the treatment or control groups during the matching process. We find that the dropped observations are not meaningfully different from either the treatment or control group. This reassures us that the matching did not lead to selectively dropping a certain subset of beneficiaries and thus upholds the generalizability of our findings.						

© 2025 Roy I et al. *JAMA Network Open*.

Sensitivity Analysis: 2021 as post-COVID year data

To address concerns about ongoing disruptions in healthcare staffing and operations during the post-COVID year 2021, we also examined pre-COVID data from 2019. Estimates from the same DiD models comparing 2015 and 2019, shown in eTable 6, are largely consistent with our main findings and do not change our conclusions.

eTable 4. *Observed Outcome Rates by Medicare Coverage Type Before Matching (n=12,543,954 total hospitalizations)*

Outcome	MA Beneficiaries			TM Beneficiaries		
	**2015**	**2019**	**2021**	**2015**	**2019**	**2021**
N	1268790	1588302	1719393	2876629	2810336	2280504
Any SNF, %	20.6	18.9	16.4	22.4	19.5	16.3
Any HHA, %	13.4	13.1	13.2	15.2	13.8	13.7
Any PAC, %	34.0	32.0	29.6	37.6	33.3	29.9
Readmission, %	22.5	22.4	23.1	23.8	23.6	24.0
Mortality, %	9.1	9.2	11.4	9.7	9.1	11.5
SNF length of stay, mean days (among those with SNF)	27.5	21.2	21.9	34.9	25.4	26.5
Improvement in activities of daily living, % (among those with SNF assessments)	19.8	23.2	22.0	19.0	21.8	19.6
HH length of stay days, mean days (among those with HH)	28.9	30.7	34.2	33.6	33.6	36.8
Improvement in activities of daily living, % (among those with HH assessments)	64.7	76.8	78.7	67.7	78.4	77.9
Days in the community, mean days	87.6	89.1	89.0	85.1	88.2	87.8

© 2025 Roy I et al. *JAMA Network Open*.

***eTable 5.** Observed Rates and Average Treatment Effect for the Treated (95% CI), MA 2015 to 2019 (n=4,605,651 hospitalizations)**

Outcome	Treatment Group MA-growth		Control Group TM		Unadjusted Estimated Effect of MA presented as linear probability (95% CI)	Doubly-Robust Adjusted Estimated Effect of MA presented as linear probability (95% CI)
	**2015**	**2019**	**2015**	**2019**		
N	539,337	729,908	1,021,114	2,315,292		
Any SNF, %	23.2	18.4	23.3	19.0	-0.5 (-1.0, 0.0)	-0.4 (-0.7, 0.0)
Any HHA, %	15.3	12.8	15.3	13.6	-0.9 (-1.5, -0.2)	-0.8 (-1.4, 0.1)
Any PAC, %	38.4	31.1	38.5	32.6	-1.4 (-2.4, -0.3)	-1.1 (-1.8, -0.5)
Readmission, %	24.7	22.0	24.2	22.9	-1.6 (-2.0, -1.3)	-1.6 (-1.9, -1.4)
Mortality, %	11.3	9.0	11.1	9.0	-0.3 (-0.5, -0.1)	-0.4 (-0.5, -0.2)
SNF length of stay, mean days (among n= 937,926 SNF stays)	35.6	20.9	35.1	25.0	-4.5 (-5.2, -3.7)	-3.6 (-4.1, -3.2)
Improvement in activities of daily living, % (among n= 746,174 SNF stays with assessments)	18.4	22.8	18.6	21.8	1.6 (1.1, 2.1)	1.3 (0.7, 1.8)
HH length of stay days, mean days (among n=645,820 HH stays)	33.9	29.9	33.5	32.9	-3.2 (-4.3, -2)	-3.0 (-4.0, -2.0)
Improvement in activities of daily living, % (among n=644,436 stays with assessments)	66.1	79.9	67.3	79.1	-0.7 (-1.4, 0.1)	-0.7 (-1.5, 0.0)
Days in the community, mean days	83.6	89.3	83.8	88.5	1.2 (0.9, 1.4)	1.2 (1.0, 1.3)

*ATT estimates are from linear difference in differences model, adjusted for variables from the propensity score model and including a fixed effect for county of residence.

© 2025 Roy I et al. *JAMA Network Open*.

Sensitivity Analysis : Composition of hospitalizations between 2015 and 2021.

To ensure composition of hospitalizations did not change differentially for MA and TM groups between 2015 and 2021, we started with describing the distribution of top 10 DRG codes across 2015 and 2021 to see if we notice differential patterns of hospitalization in MA vs TM. These descriptive data suggest that while the primary diagnosis changed over time in both groups, those changes were similar for MA and TM beneficiaries. Since change over time in the most common DRG codes was not differential (eTable 6), it should not induce bias in our estimates.

Although we feel the descriptive table supports our approach, we further wanted to confirm that patient complexity and type of hospitalization are adequately accounted for in our models. We therefore re-ran our primary models (any SNF, any HH, readmission, and mortality) with an additional adjustment for hospitalization DRG code. This additional adjustment did not impact our interpretation of results (eTable 7).

© 2025 Roy I et al. *JAMA Network Open*.

eTable 6. Distribution of Top 10 DRG Codes Across 2015 and 2021

2015

Medicare Advantage (n=1,268,790)		Percent	Traditional Medicare (n=2,876,629)		Percent
470	Hip and Knee Replacement without MCC	9.0	470	Hip and Knee Replacement without MCC	8.9
871	Septicemia or Sepsis with MCC	4.0	871	Septicemia or Sepsis with MCC	4.2
392	Digestive without MCC	2.0	392	Digestive without MCC	1.9
872	Septicemia or Sepsis without MCC	1.9	872	Septicemia or Sepsis without MCC	1.8
292	Heart Failure and Shock with CC	1.7	292	Heart Failure and Shock with CC	1.7
378	Gastrointestinal Hemorrhage with CC	1.7	378	Gastrointestinal Hemorrhage with CC	1.7
65	Intracranial Hemorrhage or Cerebral Infarction	1.6	690	Kidney and Urinary Tract Infections without MCC	1.7
291	Heart Failure and Shock with MCC	1.6	194	Pneumonia and Pleurisy with CC	1.6
683	Renal Failure with CC	1.5	291	Heart Failure and Shock with MCC	1.6
690	Kidney and Urinary Tract Infections without MCC	1.5	65	Intracranial Hemorrhage or Cerebral Infarction	1.5

2021

Medicare Advantage (n=1,719,393)		Percent	Traditional Medicare (n=2,280,504)		Percent
177	Respiratory Infections and Inflammations with MCC	6.5	871	Septicemia or Sepsis with MCC	5.8
871	Septicemia or Sepsis with MCC	6.1	177	Respiratory Infections and Inflammations with MCC	5.3
291	Heart Failure and Shock with MCC	3.7	291	Heart Failure and Shock with MCC	3.3
470	Hip and Knee Replacement without MCC	2.3	470	Hip and Knee Replacement without MCC	3.3
65	Intracranial Hemorrhage or Cerebral Infarction	1.8	872	Septicemia or Sepsis without MCC	1.7
872	Septicemia or Sepsis without MCC	1.7	65	Intracranial Hemorrhage or Cerebral Infarction	1.5
378	Gastrointestinal Hemorrhage with CC	1.5	378	Gastrointestinal Hemorrhage with CC	1.5
481	Hip and Femur Except Major Joint with CC	1.5	481	Hip and Femur Except Major Joint with CC	1.5
247	Cardiovascular Procedures with Drug-Eluting Stent without MCC	1.4	392	Digestive without MCC	1.4

© 2025 Roy I et al. *JAMA Network Open*.

683	Renal Failure with CC	1.2	247	Cardiovascular Procedures with Drug-Eluting Stent without MCC	1.3

eTable 7. *Estimation of Primary Models (Any SNF, Any HH, Readmission, and Mortality) With an Additional Adjustment for Hospitalization DRG Code*

	Adjusted estimated Effect as presented in the manuscript	*Estimated effect with additional adjustment for DRG Code*
Any SNF, n (%)	*0.8 (0.4, 1.2)*	*0.7 (0.4, 1.1)*
Any HHA, n (%)	*-0.3 (-0.8, 0.3)*	*-0.8 (-1.3, -0.3)*
Readmission, n (%)	*-1.5 (-1.8, -1.2)*	*-1.5 (-1.8, -1.3)*
Mortality, n (%)	*-0.3 (-0.6, -0.1)*	*-0.2 (-0.4, -0.0)*

© 2025 Roy I et al. *JAMA Network Open*.
